# Membrane Binding and Modulation of the PDZ Domain of PICK1

**DOI:** 10.3390/membranes5040597

**Published:** 2015-10-16

**Authors:** Simon Erlendsson, Kenneth Lindegaard Madsen

**Affiliations:** Molecular Neuropharmacology Laboratory, Lundbeck Foundation Center for Biomembranes in Nanomedicine, Department of Neuroscience and Pharmacology, Faculty of Health and Medical Sciences, The Panum Institute 18.6, University of Copenhagen, Copenhagen N 2200, Denmark; E-Mail: serlendsson@bio.ku.dk

**Keywords:** PDZ domains, lipid-binding domains, PICK1, scaffolding proteins

## Abstract

Scaffolding proteins serve to assemble protein complexes in dynamic processes by means of specific protein-protein and protein-lipid binding domains. Many of these domains bind either proteins or lipids exclusively; however, it has become increasingly evident that certain domains are capable of binding both. Especially, many PDZ domains, which are highly abundant protein-protein binding domains, bind lipids and membranes. Here we provide an overview of recent large-scale studies trying to generalize and rationalize the binding patterns as well as specificity of PDZ domains towards membrane lipids. Moreover, we review how these PDZ-membrane interactions are regulated in the case of the synaptic scaffolding protein PICK1 and how this might affect cellular localization and function.

## 1. Introduction

Scaffolding proteins are essential components required for spatial and temporal regulation of cellular processes, such as synaptic plasticity and cell-cell adhesion. The overall function of scaffolding proteins is characterized by a common ability to (I) bind its interaction partners via specific interactions using relatively short consensus motifs; and (II) bind to other cellular scaffolding proteins, mediators or specific intracellular membrane compartments. To this end, scaffolding proteins rely on both protein-protein interactions (PPIs) and protein-lipid interactions (PLIs), which can be attributed to various distinct domains or posttranslational modification (e.g., myristoylation or palmitoylation). Canonical examples of PPI domains include SRC homology 2/3 (SH2/3) domains [1,2,3,4], WW domains [5], phosphotyrosine-binding (PTB) domains [6,7], and homologous PSD-95/Disc-large (DLG1)/ZO-1 (PDZ) domains [8,9]. Prototypical membrane lipid binding domains include the phosphatidylinositide-binding Phox homology (PX) domains, Pleckstrin homology (PH) domains, C1/C2 domains, epsin N-terminal homology (ENTH) domains, and (Fab1/YotB/Vac1/EEA1) FYVE domains [10,11]. It is becoming evident, however, that many of these domains are functionally overlapping in terms of PPIs and PLIs.

The (PH) domain is one of the most prominent examples of a domain known to bind both lipid membranes and specific protein motifs [12]; however, recently several classical PPI domains have also been shown to directly interact with membrane lipids. Especially for PDZ domains, membrane lipid binding have been reported for several domains and there is accumulating evidence that the activity of PDZ domains might be tightly regulated by the spatiotemporal distribution of phosphatidylinositolphosphates (PIPs) in specific intracellular compartments [13,14].

## 2. PDZ Domain Structure and Canonical Ligand Binding

The PDZ domain is one of the most common PPI domains found in the mammalian proteome. They were first discovered in 1991 in imaginal discs of Drosophila [15] and in 1992 in rat brain homogenates [16], but was not conceptualized until 1995 [17], after a few other studies had reported the presence of “GLGF repeat” or “DHG” domains. Since then at least 266 unique PDZ domains in 150 different proteins have been identified in the human genome alone [18].

**Figure 1:**
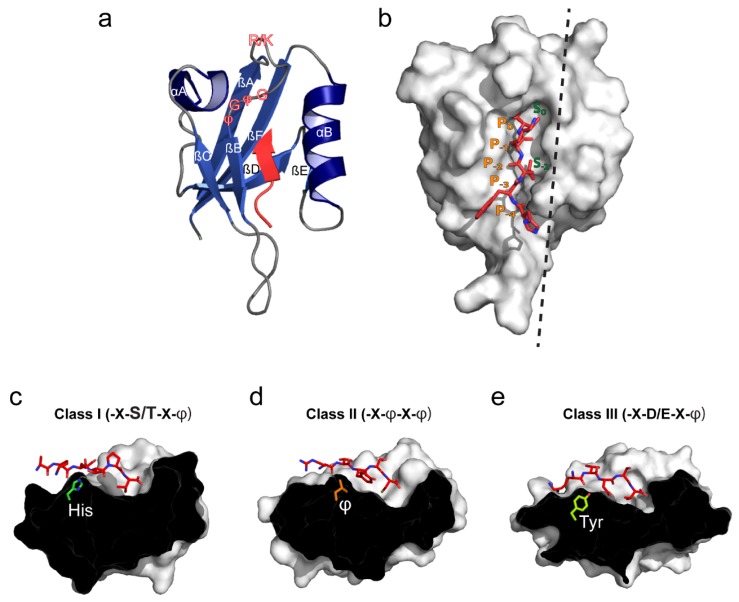
Structural overview of the canonical PDZ domain fold, ligand binding and peptide-based classification. (**a**) The canonical PDZ domain fold comprises two α-helices (αA and αB) and six β-strands (βA–βF) (PDB: 2LUI [22]). The ligand is shown in red and is stitched into the binding groove located between the αB helix and βB strand. The peptide/protein-binding groove is composed by the R/K-X-X-X-G-φ-G-φ (X—any residue; φ—hydrophobic residue) loop indicated in red outlined text; (**b**) Surface view of the canonical PDZ domain. Hydrophobic pockets S_0_ and S_-2_ (for class II domains) are indicated in green. Correspondingly, the five C-terminal ligand residues are denoted from P_0_ to P_−4_ in orange. The black dashed line indicates the cross section plane used in panels c-e; (**c**) Class I binding domains usually recognize ligands with either Ser or Thr at P_−2_. The αB1 residue is usually His (PDB: 1TP3 [23]); (**d**) Class II binding domains recognize ligands with hydrophobic residues at P_−2_. The αB1 residue is often hydrophobic and allows for formation of a second hydrophobic binding pocket (denoted S_-2_ in panel b) (PDB: 1N7F [24]); (**e**) Ligands binding class III domains typically have Asp or Glu at P_−2_ and Tyr at the αB1 position (PDB: 1B8Q [25]).

The first two studies describing the overall structural basis of PDZ domains were published in 1996. One study reported the crystal structure of the third PDZ domain (PDZ3) of PSD-95 in both a substrate-bound and a substrate-free state, whereas the other reported the substrate-free state of the homologous PDZ3 of SAP102 [9,19]. At present, 450 structures of PDZ domains are deposited in the PDB database; all sharing the same overall structural characteristics. The canonical PDZ domain is globular with an approximate diameter of 35 Å. The domain consists of ~90 residues forming a partially opened β-barrel including six β-strands (βA-βF) and two α-helices (αA and αB) capping the open sides of the β-barrel (Figure 1a). The key function of the PDZ domain is the ability to bind specific C-terminal motifs and is mediated by a confined, surface-exposed binding groove situated between the βB strand and the αB helix. The ligand is “stitched” into this binding groove, anti-parallel to the βB strand allowing for important backbone interactions. The bottom of the binding groove is constituted by a conserved R/K-X-X-X-G-φ-G-φ loop (X is any residue and φ is a hydrophobic residue) between the βA strand and the βB strand where the amides in the G-φ-G-φ motif, and possibly the basic side-chain of arginine/lysine tightly coordinate the carboxyl group of the most C-terminal residue of the ligand (denoted P_0_) [20,21] (Figure 1a,b). The actual binding groove is relatively small accommodating 3-5 C-terminal residues of the ligand, which is, in most cases, sufficient to obtain micromolar binding affinities [9,19].

Upon canonical binding the side-chain of the P_0_ residue is guided into a hydrophobic cavity constituted by residues from βB and αB of the PDZ domain (S_0_) and, consequently, the vast majority of PDZ domain ligands have a hydrophobic residue at this position. The size and shape of S_0_ varies between the different PDZ domains, thereby controlling the preference of S_0_ towards different hydrophobic residues at P_0_. Most PDZ domains, however, prefer non-aromatic hydrophobic residues at P_0_, such as valine, isoleucine or leucine [26,27,28]. As a consequence of the extended anti-parallel β-strand ligand insertion, the side chains of residues at P_−1_ and P_−3_ will be solvent-exposed and, therefore, these residues are rarely directly involved in canonical PDZ domain binding (Figure 1b). Some studies have, however, suggested that amphipathic residues such as tryptophan or tyrosine might be slightly favored at these positions [29]. In contrast, the side chain of the P_−2_ residue points towards the PDZ domain surface, like the side chain of the P_0_ residue. Most importantly, it comes in direct contact with the side chain of the first residue of αB (αB1), but also several other residues (mostly hydrophobic) from αB and βB, which constitute a secondary binding pocket (S_−2_). The preference of S-_2_ towards specific residues at P_−2_ is much stricter than the residue preference of S_0_ and, consequently, the classification of PDZ domain ligands have been primarily based on the P_−2_ residue. Correspondingly, PDZ domains have been classified with respect to the first residue of the α-helix B (αB1) [20,30,31].

The majority of PDZ domains can be readily divided into three different classes. Class I PDZ domains, which is by far the largest class, typically recognizes a -X-S/T-X-φ motif using a histidine at the αB1 position to form a hydrogen bond with the P_−2_ serine/threonine (Figure 1c) [27]. PSD-95 (PDZ2 and 3) and syntrophin are classical examples of class I PDZ domains [9,32]. Class II domains recognize -X-φ-X-φ motifs using a hydrophobic residue at the αB1 position (Figure 1d). For this reason class II PDZ domains such as CASK, GRIP1 (PDZ 5 and 6) and syntenin, are described to have a second hydrophobic pocket formed by residues at positions αB1, αB4, αB5, and βB3 [24,33,34,35]. Class III PDZ domains recognize a -X-D/E-X-φ motif and generally have a tyrosine at αB1 position, allowing the hydroxyl group to interact with the carboxyl side-chain of aspartate or glutamate (Figure 1e). The PDZ domain of nNOS typically binds class III ligands [28].

## 3. Membrane Lipid Binding Is a General Property of PDZ Domains

The membrane lipid binding capacity of Syntenin PDZ1 and 2 was the first to be described, and the mechanism, as well as the functional relevance, has been characterized [36,37,38]. Subsequently, PDZ-lipid interactions of Par3-PDZ2 [39], PTP-BAS PDZ2 [40], ZO-1 [41], and PICK1 PDZ [42] have been reported. In the lack of direct structural insight, the PDZ-membrane lipid interactions have been investigated functionally using mutational analyses in combination with biochemical assays, such as liposome sedimentation, surface plasmon resonance (on liposomes) (SPR), lipid dot-blots, and NMR. Several recent papers have reviewed the detailed structural features underlying lipid binding for a selected subset of PDZ domains [13,43]. Here we review three studies aiming to provide a general framework for understanding PDZ-membrane lipid interactions by analyzing entire PDZ domain libraries.

To address the ability of PDZ domains to interact with membrane lipids in general terms, Wu *et al.* [39] measured the liposome binding capacity by sedimentation of 74 mammalian PDZ domains. From these 74 domains, 17 were found to have detectable membrane lipid binding capacities. Furthermore, six domains, originating from five different proteins (CASK PDZ, PICK1 PDZ, X11α PDZ, DLG5-PDZ2, and ZO-1 PDZ1 and 2), were found to have liposome affinities comparable to PAR3 PDZ2. Though this study did not further investigate how the composition of the liposomes could potentially influence the membrane binding, it has been reported that most PDZ domains bind anionic membranes [39,42].

**Figure 2:**
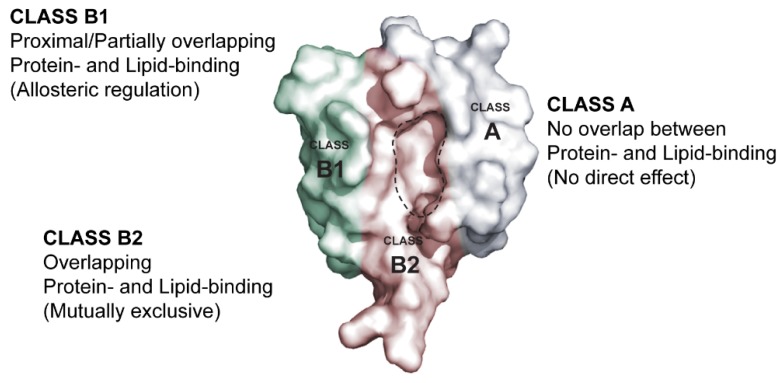
Proposed cationic patch classification of membrane lipid binding PDZ domains. The classification is based on positioning of the cationic patch as proposed by Chen *et al.* [44]. Class A domains do not display overlap of the protein and membrane lipid binding sites. The cationic patch is likely to be positioned opposite to the binding groove and/or involving the βE strand. Class B1 domains display proximal or partially overlapping protein- and membrane lipid-binding sites that may allosterically regulate each other. Often the cationic patch of class B1 domains involves residues C-terminal to the αA helix. Finally, the class B2 domains have overlapping protein- and lipid-binding sites and therefore class B2 domains will almost exclusive bind one or the other. A dashed black line delineates the binding pocket.

Another large-scale study by Chen *et al.* [44] tested membrane binding to such anionic lipids of 70 monomeric PDZ domains from 35 different mammalian proteins using SPR. Twenty-six of these were found to interact with affinities spanning three orders of magnitude (20 nM to 10 uM). Moreover, it was also stated that most of these PDZ domains did not show significant preference for specific PIPs. By using sequence information, Chen *et al.* built a prediction model using numerical vector representation as a function of primary and tertiary structure combined with residue specific information from which they predicted membrane binding of 2000 PDZ domains from 20 different species. They found that most membrane lipid binding PDZ domains have at least one cationic site. The position of the cationic site was subsequently used to divide PDZ domains into three distinct classes (Figure 2). Class A domains have a cationic site not overlapping with the peptide binding groove, whereas class B domains have a cationic site proximal to the peptide binding groove, typically clustered around the αA helix. This class was subdivided into classes B1 and B2. B1 domains typically have the cationic patch conserved at the C-terminal end of αA helix and are, therefore, able to simultaneously interact with peptides and membrane lipids. As an example, the presence of plasma membrane mimetic liposomes allosterically alters peptide binding for rhophillin2 PDZ. B2 domains, on the other hand, display overlap between the peptide binding groove and the membrane lipid binding cationic patch. As an example, binding to plasma membrane mimetic liposomes significantly reduced the binding to peptide ligands for tamalin PDZ. B2 domains seem to have the cationic patch conserved at the N-terminal end or scattered throughout the αA helix.

Finally, a recent study sought to generalize the PDZ domain lipid binding and the nature of PDZ/peptide/lipid complexes using a fluorescence-based cell-localization assay [45]. Out of 246 investigated single domains, 53 displayed distinct cellular localization; either with discrete or strong plasma membrane localization or localization to cytosolic, or subnuclear organelles. Most PDZ domains displayed either discrete plasma membrane localization or localized to subnuclear organelles. Very few showed strong plasma membrane or cytosolic localization. Interestingly, localization was strongly cell-type dependent, which was hypothesized to be due to differences in ligand expression or lipid composition. It was further tested whether changes in the specific PIP composition of relevant cellular compartments would in fact also drive de- or relocalization of the PDZ domains. Neither strong and discrete plasma membrane localized PDZ domains showed significant PIP dependency. In fact, only the PDZ domain of CASK displayed sensitivity towards differences in the PIP plasma membrane composition. For the subnuclear localized domains; however, three out of six randomly-selected domains, including the deafness, autosomal recessive 31 PDZ1 (DFNB31_1), the sodium-hydrogen exchange regulatory cofactor NHERF2 PDZ1 (SLC9A3R2_1) and the Syntrophin, Gamma 1 PDZ domain (SNTG1) showed PI(4,5)P dependency, and displayed a more diffuse nuclear or cytoplasmic localization upon membrane lipid modifying treatments. The cytosolic PDZ domains, primarily localized to perioxisomes or appeared as cytosolic aggregates and showed no clear PIP dependency.

To investigate if the subcellular localization could be explained directly by specificity towards different PIPs, the affinities for liposomes with different PIP contents were quantified using SPR. Out of 19 randomly selected domains, 10 domains displayed micromolar affinities towards tri- or biphosphorylated PIPs. Only one PDZ domain displayed micromolar affinity for monophosphorylated PIPs (DFNB31_1). Importantly, all of the four domains found to respond to cellular lipid modification also displayed PIP specificity. The authors further tested the affinity towards several other membrane lipids (e.g., cardiolipin, phosphatidylserine (PS), phospatidylcholine (PC), and cholesterol, *etc.*) using a lipid dot blot assay. This assay revealed that a few domains also have the ability to bind non-phosphorylated lipids. Interestingly, the PIP affinities determined from the SPR experiments was different from affinities obtained using dot blots, suggesting that some of these interactions relies on the presence of intact membranes. In agreement with findings by Chen *et al.* and adding to the general understanding of PDZ-membrane lipid interactions, it was suggested that high pI (>9) seems to be a common feature of the lipid binding PDZ domains, and that conserved cationic patches are important for PIP specificity and cellular localization [45].

Though it has become well established that lipid/membrane binding is a common feature shared by several PDZ domains, the molecular mechanisms underlying the binding are still poorly understood. As presented above, attempts to classify the domains according to either their cellular localization, or their cationic properties, have provided a valuable framework for understanding how these interactions might be electrostatically favored. To emphasize how these general properties may relate to regulation by e.g., redox state, ion binding, and posttranslational modifications we review the case of the lipid and membrane binding by the PICK1 PDZ domain below.

## 4. PICK1 Is a Flexible Membrane-Associated Scaffolding Protein

Protein Interacting with C kinase 1 (PICK1) is ~47 kDa membrane associated scaffolding protein which was first discovered in a yeast two hybrid experiment investigating interaction partners of activated Protein Kinase C (PKC) [46]. The overall size varies from 400 to 500 aa across different species with the largest difference being found in the length of the C-termini [47]. In vertebrates, PICK1 is highly expressed in the brain (detected in high levels in Cerebellar Purkinje neurons, and hippocampal neurons), in the pancreas, and in the testis. Additionally, heart, lung, spleen, liver, and muscle tissue were found to contain modest levels of PICK1 [48,49].

Functionally, PICK1 plays a pivotal role in regulation of synaptic plasticity involving trafficking of the AMPA-type ionotropic glutamate receptors (AMPARs), which is thought to be the neuronal basis for learning and memory [50]. PICK1 interacts directly with the GluA2 subunit of the AMPAR via its PDZ domain, and has been shown to promote activity-stimulated AMPAR internalization [51]. Moreover, PICK1 reduces the recycling rate of internalized AMPARs giving rise to an overall intracellular accumulation [52,53]. As a result of these separate functions PICK1 is necessary for long-term depression [49], as well as some types of long term potentiation of neuronal synapses [54]. Through the functional interaction with the AMPARs PICK1 is, moreover, implicated in the pathogenesis of several psychiatric and neurological disorders [55] and is, consequently, considered a promising novel drug target. More recently, PICK1 has been shown to associate with the Golgi compartment and regulate the biogenesis of dense core vesicles in the pancreas, pituitary, and adrenal glands, thereby affecting metabolic homeostasis in flies and mice [56,57,58].

At the structural level, PICK1 contains two highly conserved domains; the ~90 aa N-terminal PDZ domain and by a ~210 aa Bin/amphipysin/Rvs (BAR) domain which mediates strong homo-dimerization. PICK1 is the only protein featuring these two domains and they are separated by a ~40 aa linker region capable of forming a four-turn membrane binding amphipatic helix (AH). The domains and are flanked by a ~20 aa acidic N-terminal region (NAR) and a ~50 aa acidic C-terminal region (CAR). The PICK1 homo-dimers adopt, overall, an elongated crescent shape defined by the BAR domain [59,60,61] which form a coiled-coil six helix bundle upon dimerization as seen for *i.e.*, amphiphysin, arfaptin, and endophilin [62,63,64]. Two recent studies utilized small angle X-ray scattering (SAXS) to determine the entire quaternary structure of the PICK1 homo-dimer [60,61]. The studies largely agree on the overall structure of the BAR domain and the dimerization interface, but differ considerably with respect to the positioning of the PDZ domains relative to the BAR domain. Madasu *et al.* suggest that the PDZ domains are associated with the BAR domain and the linker between the adjacent domain forms a permanent helix [61], whereas we suggest the PDZ domain to be flexibly attached through an unstructured linker that only folds into an amphipathic helix in the presence of membrane binding [60]. This controversy is central to the understanding of PICK1 function, since the PDZ domain has been suggested to auto-inhibit the membrane binding capacity of the BAR domain through steric hindrance [53,65,66,67].

## 5. PICK1 Contains a Non-Canonical Promiscuous PDZ Domain

Each monomer of PICK1 contains a single PDZ domain, which is highly conserved between species from mollusks to vertebrates. The PDZ domain of PICK1 interacts with more than 40 different ligands, including GluA2, the dopamine transporter (DAT), mGluR7, Glt1b, and ASIC1a through their respective C-termini [22,47,68,69,70]. Remarkably, these ligands belong both to class I and II and several ligands fall entirely outside the classification. Only a few other PDZ domains, e.g., PAR3-PDZ3[71], have been reported to bind ligands of distinct classes. Though the PICK1 PDZ domain is indeed highly promiscuous, class II ligands are preferred over class I and non-classified ligands. The binding affinity of the PICK1-PDZ domain with this class II distal C-terminus of DAT (0.8 μM) is almost 20-fold better than the interaction between PICK1-PDZ and the Protein Kinase C α (PKCα) class I C-terminus (14 μM) [22].

**Figure 3:**
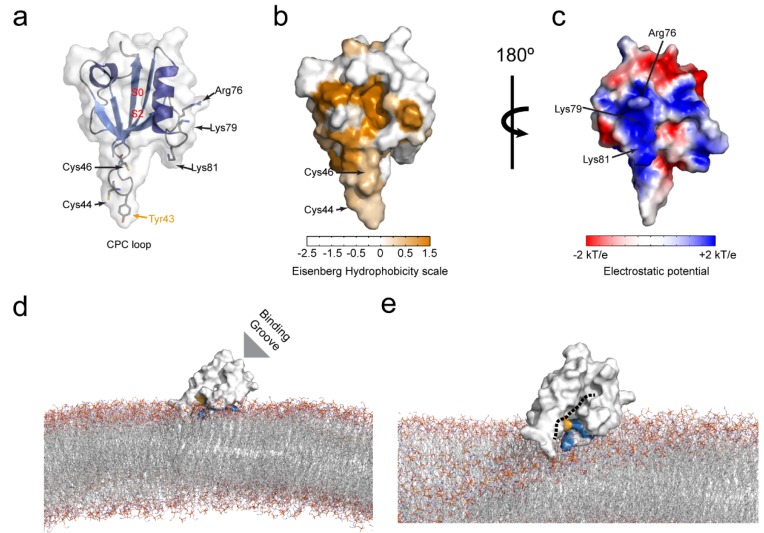
Molecular determinants of the PICK1 PDZ domain lipid membrane binding. (**a**) Cartoon and surface view of the PICK1 PDZ (PDB: 2LUI) [22] domain. Hydrophobic pocket I and II are depicted by S0 and S2, respectively. Cys44 and Cys46 in the CPC loop are indicated by arrows and shown as sticks. Arg76, Lys79, and Lys81, comprising the cationic patch, juxtapose to the canonical binding groove are also indicated by arrows and shown as sticks. Tyr43 involved in the upstream peptide-binding mode in indicated in orange; (**b**) The PICK1 PDZ domain colored by hydrophobicity (Eisenberg scale). Evidently, the binding pocket is highly hydrophobic, but also the CPC loop is predominantly hydrophobic; (**c**) The cationic patch is clearly visible when calculating and displaying the electrostatic potential of the domain; (**d**) Proposed orientation of the PICK PDZ domain in context of a negatively charged membrane. When the hydrophobic CPC loop is partly inserted into the membrane (involving one or both Cys residues, as well as Tyr43) and the cationic patch (shown in blue) facing the negatively charged membrane surface, the binding groove will still be completely accessible; (**e**) A rotated view of the proposed PICK1 PDZ membrane interaction suggests that the binding pocket (indicated by dashed black line) is optimally positioned to bind the C-termini of membrane embedded ligands.

The isolated PICK1 PDZ domain has been fully structurally characterized using both X-ray crystallography and NMR spectroscopy, in complex with canonical class II ligands; DAT, GluA2, and EphrinB1. The truncated PDZ domain is only stable upon direct fusion to ligands which can effectively occupy the PDZ binding groove, however, the canonical overall globular fold consisting of six β-strands and two α-helices is conserved [22,42,72,73]. For these class II ligands the interaction is primarily facilitated via two hydrophobic pockets, S_0_ and S_2_ (see Figure 1b and Figure 3a). Hydrophobic pocket I (S_0_) consists of the G-φ-G-φ motif involving Ile33, Gly34, Ile35 but also a conserved Ile90 at position αB8. A preference for P_0_ residues of Val > Ile > Leu was previously found for S_0_ [68]. Hydrophobic pocket II (S_2_) consists of residues at positions αB1, αB4, αB5 and βB3; Lys83, Val86, Ala87, and Ile37. Notably, the αB1 residue is a highly conserved Lysine (Lys83), which is unique for the PICK1 PDZ domain. The amine side-chain of Lys83 can interact with the peptide backbone in P_−4_ of the ligand, and the aliphatic chain can complement the hydrophobic pocket II to make a favorable environment for a hydrophobic residue at P_-2_ in class II ligands [72]. The bottom of the PICK1-PDZ binding groove contains a conserved lysine (Lys27) that in the crystal structures has been shown to interact through a highly ordered water molecule, with the carboxyl C-terminal of the ligand [72].

PICK1 PDZ promiscuity relies on a combination of increased tolerance in the canonical binding pocket and additional non-canonical binding motifs [22]. Thus, the interaction with Class I ligand, PKCα, depends on residues upstream from the canonical binding sequence, which are likely to interact with a flexible loop between βB and βC (Tyr43 in particular, see Figure 3a) of the PDZ domain. Several additional ligands (e.g., GluA2, HER2, mGluR7b, UNC5H1, Glt1b) have bulky hydrophobic residues in the P_-7_ position, which may favor interaction with this part of the PICK1 PDZ domain. Furthermore, it was demonstrated that the unconventional ligand ASIC1a has a dual binding mode involving a canonical insertion and a non-canonical internal insertion with the two most C-terminal residues (P_0_ and P_-1_) forming interactions outside the groove.

## 6. Membrane Lipid Binding of the PICK1 PDZ Domain

### 6.1. Molecular Determinants of the PICK1 PDZ Domain Membrane Lipid Binding

The first studies of the membrane lipid binding capacities of PICK1 were focused exclusively on the BAR domain and suggested that the involvement of the PDZ domains was primarily to regulate the accessibility of the BAR domain to the membranes [49,53,65,66,67]. Nevertheless, truncation studies suggested that the PDZ domain rather reinforces the membrane binding capability of the BAR domain [66].

Direct binding studies with the truncated PDZ domain stabilized with the self-binding GluA2 peptide confirmed membrane lipid binding both on dot blots and in context of membranes [42]. Using lipid dot-blots it was found that PICK1 PDZ binds weakly to PI3P, PI4P, PI5P, PI(3,5)P, PI(4,5)P, and PI(3,4,5)P compared to the stronger binding observed for the PAR3 PDZ2 domain. These findings were supported by liposome sedimentation studies where PICK1 PDZ displayed dose-dependent interaction with bovine brain liposomes with an apparent Kd of 3.6 ± 0.5 ug/mg. To specifically probe the preference towards different PIPs, PC/PS (80/20) liposomes containing 10% PI3P, PI(4,5)P or PI(3,4,5)P were also tested. Consistent with the findings in the lipid dot-blot assay, PICK1 PDZ displayed preference towards the various PIPs in the following order: PI(3,4,5)P > PI(4,5)P ≥ PI3P. Interestingly it was shown that PICK1 seems to also weakly bind to PC/PS liposomes. By strategic mutations it was demonstrated that the lipid binding capacity of PICK PDZ towards bovine brain liposomes was mediated by two distinct binding motifs: a cationic patch on the opposite site of the PICK1 PDZ binding groove involving three positively charged residues, Arg76, Lys79 and Lys81 (Figure 3a,c), and a flexible loop between βB and βC involving a conserved CPC (Cys44-Pro45-Cys46) motif (Figure 3a,b). Mutation of Lys79 and Lys81 residues in the cationic patch into either Ala or Glu (K79/81A,E) significantly compromised liposome binding, without fully eliminating it. Mutation of both or individual Cys residues (C44G, C46G) in the CPC motif, on the other hand, completely abolished the PICK1 PDZ liposome binding capacity. The importance of the CPC loop was further substantiated by chemical modification of the cysteine residues into S-carboxymethyl-cysteine using iodoacetic acid, which similarly disrupted liposome binding. Functional studies furthermore demonstrated that mutation of the CPC loop compromised synaptic localization of PICK1 in hippocampal neurons, as well as the role of PICK1 in regulation of AMPAR trafficking. More recently, we further demonstrated that mutation of the CPC loop likewise compromised the PICK1 function in biogenesis of dense core vesicles in chromafin cells from the adrenal gland [74].

It was previously shown for the lipid binding of the PAR3-PDZ2 domain how a hydrophobic motif was inserted into the cytosolic bilayer of the membrane, thereby stabilizing the interaction [39]. To test whether the CPC loop of PICK1 PDZ could rely on the same insertion-based mechanism, the accessibility of the Cys residue was probed using Ellmans reagent (DTNB). The accessibility of the Cys residues was slightly reduced in presence of PC/PS liposomes but significantly reduced in presence of PI(4,5)P liposomes for the wild-type protein. In contrast, both the C44G and the C46G mutation introduced separately allowed full accessibility of the remaining Cys residue. Interestingly, the K79/81A,E mutations caused an increase in the Cys accessibility in presence of PI(4,5)P liposomes, but not in presence of PC/PS liposomes. Together these findings suggested that the cationic patch might serve to recruit the PICK1 PDZ domain to PIP–enriched liposomes via non-specific electrostatic interactions but that the PICK1 PDZ domain liposome binding is facilitated via an insertion-based mechanism, dependent on the two aliphatic cysteine side chains in the CPC loop. Furthermore, in all of the above experiments, the PICK1 PDZ domain was fused to the GluA2 C-terminus in order to stabilize it. This suggests that peptide binding and lipid binding are not mutually exclusive which concurs with the position of the cationic patch (Figure 3c), making the domain a typical class A membrane lipid-binding PDZ domain. We note that Tyr43, which is important for binding of the PKCα C-terminal peptide and likely several other ligands, is probably embedded in the lipid bilayer together with the CPC loop upon membrane binding of the PDZ domain (Figure 3a). However, the interplay between PDZ membrane and peptide binding has not yet been directly addressed experimentally (Figure 3d,e).

### 6.2. The CPC Loop of PICK1 PDZ Is a Proposed Zinc Binding Site

**Figure 4:**
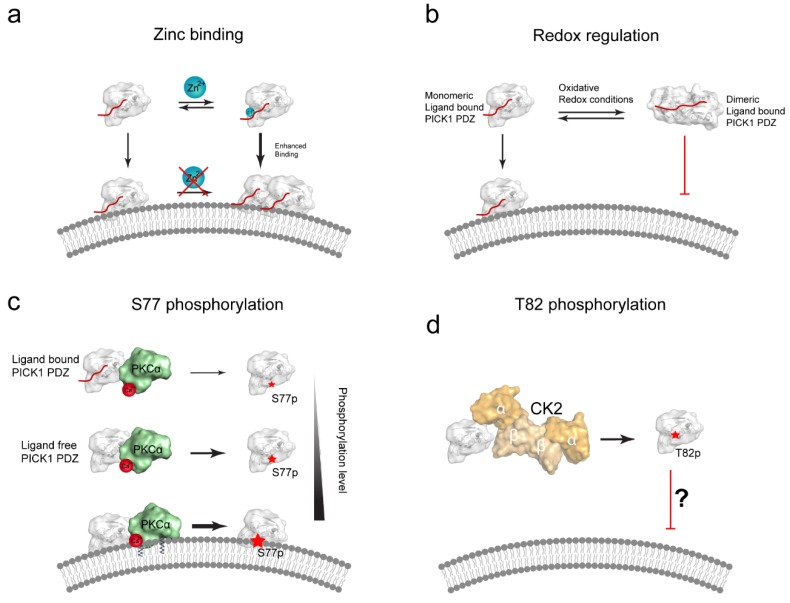
Proposed regulatory mechanisms for the PICK1 PDZ membrane binding. (**a**) Zn^2+^-binding has been suggested to positively regulate the membrane binding of the isolated, ligand bound PICK1 PDZ domain. This interaction was shown to involve the Cys44 and Cys46 in the CPC loop. Remarkably, Zn^2+^-binding could be completely abolished when the PDZ domain was already bound to liposomes, suggesting that this mechanism does not require/allow Zn^2+^ to be inserted into the membrane [78]; (**b**) Proposed mechanism for redox regulation of the PICK1 PDZ domain. PICK1 forms reversible dimeric complexes under oxidative conditions, via interdomain cystines involving Cys44 and Cys46. In contrast to the monomeric ligand bound (ligand shown in red) PDZ domain, the dimeric ligand bound PDZ domain complex is not capable of binding liposomes (Dimeric PICK1-PDZ PDB:3HPK) [73]; (**c**) Ser77, positioned in the cationic patch, is a PKCα (shown in green, PDB: 3GPE [79]) substrate [77]. Phosphorylation of Ser77 in full length PICK1 was decreased in presence of a PDZ domain ligand, compared to a ligand free PDZ domain. Conversely, the phosphorylation level was significantly enhanced in the presence of liposomes. A decrease in clustering in COS7 cells suggested that the S77D phospho-mimetic could weaken the PDZ:lipid interaction in cells; (**d**) T82, positioned at the edge of the binding groove, is a putative phosphorylation site for CK2 (shown in shades of orange, PDB: 1JWH [80]). T82E likewise displays decrease cellular PICK1:GluA2 co-clusters, in a mechanism possible alleviating subcellular localization by reduced PDZ domain lipid binding capacity.

Interestingly, CPC motifs are conserved in P_1B_ ATPases, where they localize to the center of the sixth transmembrane helix (TM6) and are directly involved in coordinating both mono- and bivalent cations (e.g., Cu^+^, Cu^2+^, Cd^2+^ and Zn^2+^) during transport [75]. Using fluorescence spectroscopy Shi *et al.* showed that Zn^2+^ can also bind specifically to the CPC loop of the PICK1 PDZ domain covalently conjugated to the GluA2 C-terminus [76]. The stoichiometry was determined to be 1:1 and an association constant to be 8.17 ± 0.23 × 10^5^/M. The *K_d_* was estimated to be in the lower micromolar range. Although zinc was not found to be necessary for retaining PICK1 PDZ liposome binding, it was found that Zn^2+^ significantly enhanced the liposome binding capacity of PICK1 PDZ, but that the interaction between liposomes and PICK1 PDZ decreased the ability of PICK1 PDZ to bind zinc. It was hypothesized that the Zn^2+^-binding might increase liposome binding by stabilization of the interaction itself or by stimulation of a PDZ/PDZ dimer (Figure 4a). The PDZ dimer, however, was later shown to have compromised liposome binding (see next paragraph) [73].

### 6.3. PICK1 Membrane Binding May by Redox-Regulated

A study from Shi *et al.* [73] showed that the CPC loop Cys residues form inter-domain disulphide bonds under mild oxidizing conditions both *in vitro* and in cells under oxidative stress and thereby facilitate stable covalently linked PICK1 PDZ domain dimer complexes. This dimeric PDZ arrangement allowed Shi *et al.* to solve a dimeric PICK1-PDZ crystal structure. Interestingly the authors also showed that the domain dimerization significantly reduced the GluA2 peptide binding affinity and, furthermore, completely obliterated the liposome binding capacity of the PICK1 PDZ domain. On this basis it was hypothesized that mild oxidation of one or both of the Cys residues could be directly involved in regulating the ability of the PICK1 PDZ domain to bind lipid membranes (Figure 4b). However, it remains to be determined to which extent such oxidative conditions and consequently the modifications are relevant *in vivo*.

### 6.4. Posttranslational Modifications of PICK1 PDZ Domain Can Regulate Membrane Binding

Posttranslational modifications (PTMs) might also play an important role in regulating the membrane lipid binding of the PICK1 PDZ domain. Ser77, which is part of the proposed lipid-binding motif opposite the binding groove, is particularly interesting. Recently, Ammendrup-Johnsen *et al.* [77], showed that Ser77 is phosphorylated by PKCα. Though the C-terminus of PKCα interacts with the PICK1 PDZ domain ligand [46] the phosphorylation level did not depend on a direct PDZ-PKCα interaction, nor did the phospho-mimetic mutant, S77D, alter PDZ affinities in solution. On the contrary, it was found that PDZ domain ligand binding slightly decreased the phosphorylation level of Ser77.

In presence of liposomes the phosphorylation level of Ser77 by PKCα increased a staggering 10-fold. As PKCα is also capable of binding to lipid membranes [81], it was rationalized that the membrane could act as a scaffold to facilitate phosphorylation by bringing both PKCα and PICK1 in proximity to the membrane (Figure 4c). Since Ser77 is positioned in the actual cationic patch of the PICK1 PDZ domain it is interesting that membrane binding does not sterically hinder the phosphorylation but rather promotes it. This could simply be due to the fact that dynamics/flexibility is still sufficient to allow Ser77 to access the catalytic site of PKCα even on the membrane, or it could perhaps suggest a hitherto unseen alternative insertion mechanism. Although S77D did not affect PICK1 membrane binding, as determined by the liposome sedimentation assay, it can be speculated that the functional role of this phosphorylation might be to facilitate dissociation of PICK1 from the membrane and subsequently facilitate its cellular redistribution. This hypothesis remains to be tested.

Finally, Thr82, which is situated at the edge of the binding pocket, has been predicted to be a potential Casein Kinase (CK2) phosphorylation site. The effect of this particular phosphorylation, was recently investigated by using the PICK1 T82E mutation phospho-mimetic [78]. In this study it was found that PICK1 T82E decreased the cellular co-clustering (not the co-localization, as such) with GluA2 and also failed to regulate its trafficking. Interestingly, the T82E mutation does not seem to directly affect the binding of the GluA2 C-terminus *in vitro (Erlendsson, Madsen, unpublished)* suggesting that the redistribution from punctate PICK1-GluA2 co-clusters into more diffuse cellular co-localization might be controlled by an altered ability of PICK1 T82E to bind specific subcellular compartments (Figure 4d). It is still unclear under which conditions and by which kinase T82 is actually phosphorylated.

## 7. Future Directions

The work reviewed here suggests that a general consensus regarding the mechanisms underlying lipid/membrane binding by PDZ domains is developing—yet direct structural evidence is still lacking and general conclusions might be compromised by the methodological gap between SPR based approaches, dot-blots and steady state cellular studies using confocal microscopy. The exact binding mechanism is likely to differ between domains; however, we review two prevailing mechanisms that have been shown to facilitate PDZ-membrane lipid interactions. Interestingly, and possibly important for cellular localization, several PDZ domains have different specificity towards negatively charged lipids (*i.e.*, PIPs), which are prominent in the cytosolic leaflets of cellular membranes. This feature seems to rely on a surface-exposed cationic patch. The positioning of the cationic patch with respect to the peptide-binding groove differs between domains and, therefore, membrane binding also affects PDZ peptide/protein binding very differently. Another mechanism that has not been yet been described in general terms, is the insertion of hydrophobic appendices into the leaflet of the membrane bilayer as seen for the PDZ domain of PICK. Recent findings suggest that such insertion based membrane binding share several common features, including high functional affinity, slow kinetics, and membrane curvature sensing [82]. It will be of great interest to see whether this also applies to the membrane binding of PDZ domains and how these features relate to the function of full-length scaffolding proteins.
